# Exercise testing in patients with tricuspid regurgitation undergoing transcatheter tricuspid valve intervention

**DOI:** 10.1007/s00392-024-02554-8

**Published:** 2024-10-09

**Authors:** Muhammed Gerçek, Maria Ivannikova, Arseniy Goncharov, Mustafa Gerçek, Maximilian Mörsdorf, Johannes Kirchner, Felix Rudolph, Tanja K. Rudolph, Volker Rudolph, Kai P. Friedrichs, Daniel Dumitrescu

**Affiliations:** 1 Clinic for General and Interventional Cardiology/Angiology, Herz- Und Diabeteszentrum NRW, Ruhr-Universität Bochum, Med. Fakultät OWL (Universität Bielefeld), Georgstraße 11, 32545 Bad Oeynhausen, Germany; 2 Clinic for Cardiac Surgery and Pediatric Cardiac Surgery, Heart Center Duisburg, Duisburg, Germany

**Keywords:** Tricuspid regurgitation, Transcatheter therapy, Cardiopulmonary exercise testing, Constant work-rate exercise testing, Transcatheter edge-to-edge repair, Transcatheter tricuspid valve annuloplasty

## Abstract

**Background:**

Transcatheter tricuspid valve intervention (TTVI) has shown promising results with persistent reduction of tricuspid regurgitation (TR) and improvements in functional class and quality of life (QOL).

**Objectives:**

To analyze the impact of TTVI on maximal and submaximal exercise capacity (SEC).

**Methods:**

Constant work-rate exercise-time (CWRET) testing reflects SEC, which is more likely to be relevant for daily life activities and provides more differentiated physiological insight into the nature of exercise intolerance. Thus, 30 patients undergoing TTVI (21 direct annuloplasty and 9 edge-to-edge repair) received cardiopulmonary exercise testing (CPET) and CWRET (at 75% of maximum work rate in the initial CPET) before and 3 months after TTVI.

**Results:**

Patients’ age was 80.5 [74.8–82.3] years and 53.3% were female. TR reduction ≥ 2 grades was achieved in 93.3% (TR grade ≤ moderate in 83.3%). Echocardiography revealed improved right ventricular (RV) characteristics with decreased RV basal diameter (47.0 mm [43.0–54.3] vs. 41.5 mm [36.8–48.0]; *p* < 0.001) and decreased inferior caval vein diameter. CWRET testing showed a significantly improved SEC (246.5 s [153.8–416.8] vs. 338.5 s [238.8–611.8] *p* = 0.001). Maximum oxygen uptake showed a positive trend without statistically significant differences (9.9 ml/min/kg [8.6–12.4] vs. 11.7 ml/min/kg [9.7–13.3]; *p* = 0.31). In contrast to the six-minute-walking distance (6MWD), SEC correlated moderately with effective regurgitation orifice area reduction (*r* = 0.385; *p* = 0.036), increased cardiac output (*r* = 0.378; *p* = 0.039), and improved QOL (*r* = 387; *p* = 0.035).

**Conclusion:**

Improvements in exercise capacity after TTVI mainly occur in the submaximal rather than in the maximal exercise range and correlate with hemodynamic effects and QOL. This may have a methodological impact on assessment of exercise capacity in these patients.

**Graphical abstract:**

Improvements in exercise capacity mainly occur in the submaximal (Constant work-rate exercise-time, CWRET) rather than in the maximal exercise range (maximum oxygen consumption, peak *V*O_2_), and correlate with reduction in tricuspid regurgitation, hemodynamic effects and QOL

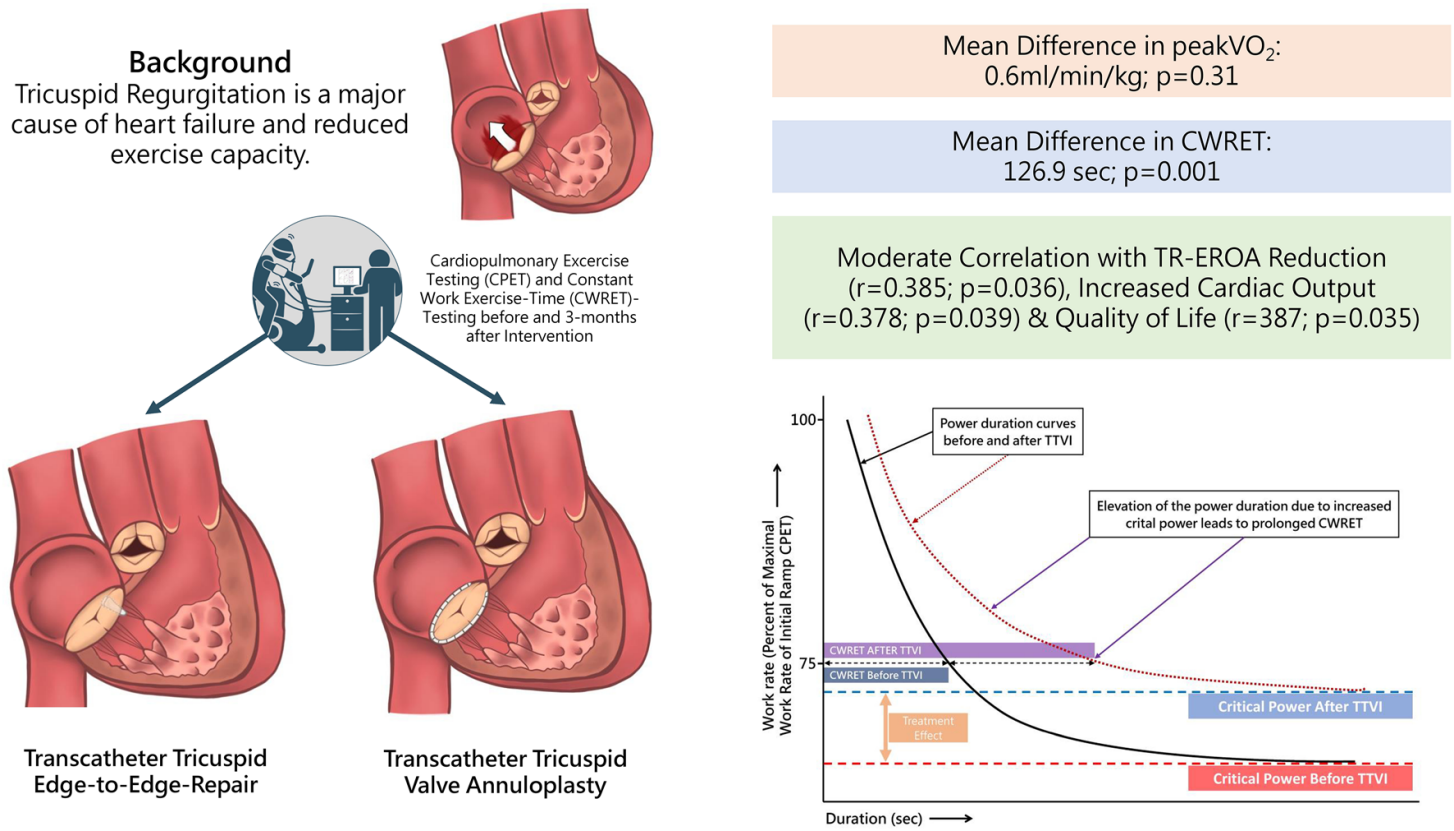

**Supplementary Information:**

The online version contains supplementary material available at 10.1007/s00392-024-02554-8.

## Introduction

Tricuspid regurgitation (TR) is a major cause of heart failure with impaired clinical outcome including reduced exercise capacity as well as impaired quality of life (QOL) [1, 2] and negatively affects patients’ prognosis [3]. Drug-based therapy is increasingly ineffective in advanced disease stages, while surgery is associated with high in-hospital mortality [4, 5]. Thus, to address this unmet need for treatment strategies, transcatheter tricuspid valve interventions (TTVI) have become one of the most dynamic fields in interventional cardiology. To date, transcatheter edge-to-edge repair (TEER) and direct annuloplasty are the most favored interventional treatment strategies, and are showing promising results with sustained reduction of TR and clinical improvements such as increased exercise capacity in the six-minute walking distance (6MWD) and improved QOL [6].

Impaired cardiopulmonary exercise capacity is known as an independent predictor of mortality in heart failure patients [7]. Accordingly, impaired functional capacity in the 6MWD has been highlighted to be of prognostic impact in patients with severe mitral regurgitation having undergone transcatheter mitral valve repair [8].

**Fig. 1:**
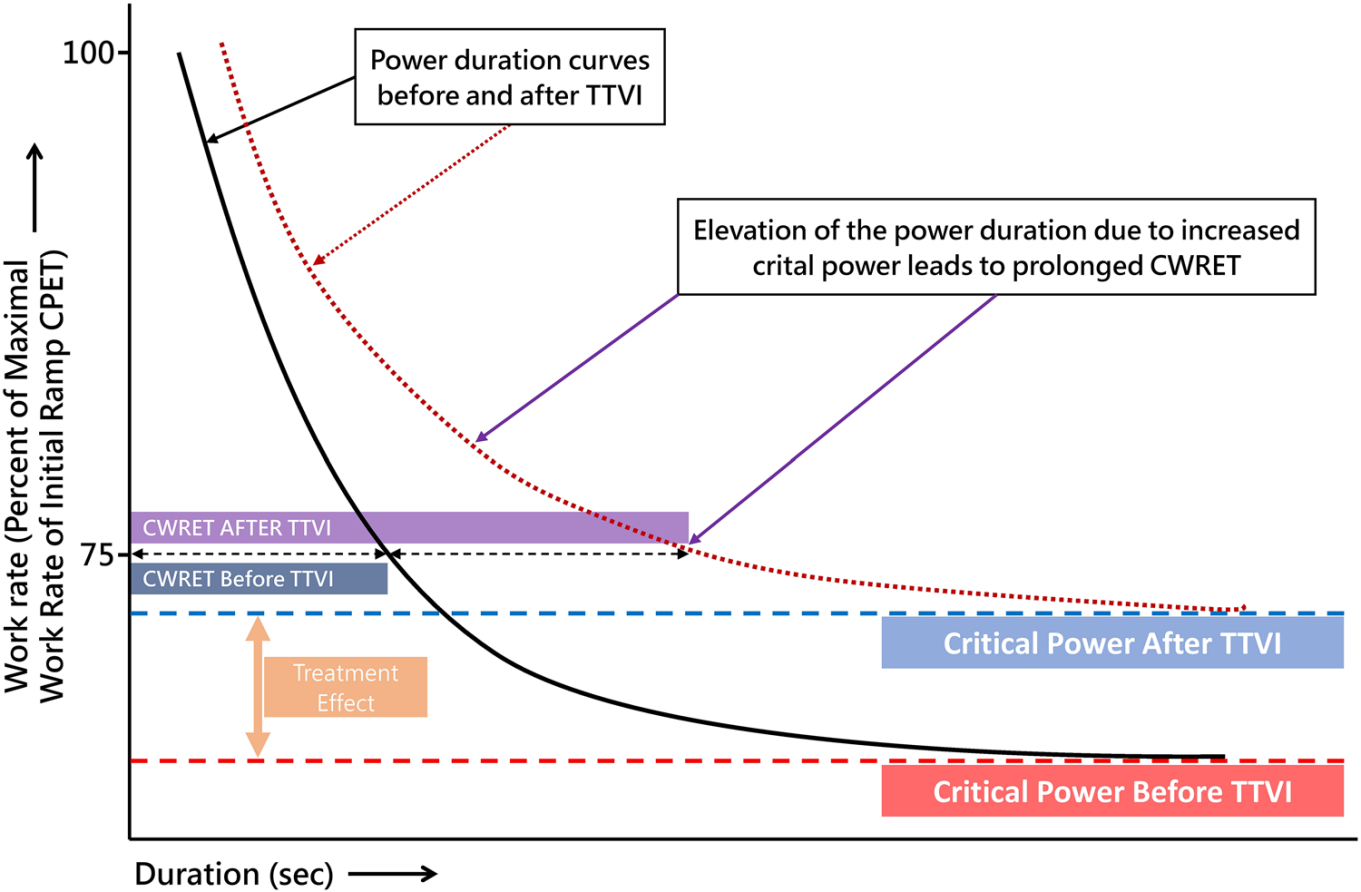
Constant work-rate exercise time (CWRET) testing. CWRET reflects submaximal/endurance exercise capacity, which is more likely to be relevant for daily life activities. Schematic representation, modified from Whipp et al. [10]. Based on these physiological considerations, treatment effects will primarily affect submaximal exercise capacity, and only lead to minor changes in maximal exercise tolerance. However, even small potential shifts in the power–duration curve will have a more pronounced impact on critical power, which is the highest individual constant workload on the power–duration curve that can be sustained in a steady-state without a potential time limit. CWRET correlates with critical power, and reflects true aerobic capacity. Thus, treatment effects in the submaximal exercise range will primarily be reflected by a significantly prolonged CWRET, even when only minor changes in maximal exercise capacity occur. *CPET* cardiopulmonary exercise test, *TTVI* transcatheter tricuspid valve intervention

However, less is known about the impact of exercise capacity on the outcome in patients with severe TR as the predominant cause of heart failure. The 6MWD is a widely established method to assess patients’ functional capacity and has the advantage of easy administration and minimal cost, but it offers only limited insight on mechanisms of exercise limitation or of treatment effects. Cardiopulmonary exercise testing (CPET) is probably the most comprehensive testing approach, which enables evaluation of the pulmonary, cardiovascular, muscular, and cellular oxidative systems [7, 9]. Due to these prognostic implications, CPET has evolved into a widely used application in cardiology especially in the setting of heart failure [9]. Yet, systematic CPET analysis in patients with TR is lacking and may provide a distinctive insight on clinical outcome and characterize functional improvements more precisely. Therefore, this study aims to analyze CPET performances in patients with symptomatic severe TR before TTVI and in follow-up examinations. To distinguish improvements in submaximal exercise endurance/capacity (SEC) from those in the maximal exercise capacity range, constant work-rate exercise time testing (CWRET) was performed in addition to standardized incremental ramp tests. As a submaximal exercise test, CWRET is a reflection of the critical power concept [10]. It is based on the power–duration relationship, which shows the amount of time that an individual subject or patient is able to sustain exercise at any given constant workload. Therefore, the power–duration curve has a hyperbolic shape (schematically shown in Fig. 1). Heavy exercise can only be sustained for a short time. With decreasing workloads, subjects will be able to sustain exercise for a longer period of time. Critical power is the highest individual constant workload on the power–duration curve that can be sustained in a steady-state without a potential time limit, and therefore reflects true aerobic capacity. The critical power concept addresses the challenge that treatment effects rather lead to improvements in the submaximal exercise range, and not maximal exercise capacity. According to Fig. 1, even slight improvements in peak exercise capacity and small effects to the power–duration curve may lead to large improvements in exercise time at submaximal constant workloads. Thus, compared to an incremental exercise test, CWRET is more likely to reflect daily life activities and is, hence, more suitable for the quantification of treatment effects [11] and providing a more nuanced physiological insight into the nature of exercise intolerance [12]. Therefore, we hypothesized that CWRET would be able to detect an intervention effect after TTVI in the submaximal individual exercise range, independent from changes in maximal exercise capacity.

## Methods

### Study population

Consecutive patients, who were not receiving inotropics, and who were able to ride a bicycle with at least severe functional TR and symptomatic heart failure (NYHA ≥ II) and who had undergone TTVI using direct annuloplasty employing the Cardioband tricuspid valve reconstruction system (Edwards Lifesciences, Irvine, CA) or with an edge-to-edge device using the TriClip (Abbott, Chicago, IL) or the PASCAL transcatheter valve repair system (Edwards Lifesciences, Irvine, CA) underwent CPET before and 3 months after the procedure between December 2019 and April 2022. Furthermore, CWRET was performed in addition to an incremental ramp CPET, the 6MWD, and QOL assessment using the Minnesota Living with Heart Failure Questionnaire (MLHFQ). Echocardiographic evaluations were performed by highly qualified medical staff following the recommendations of the American and European Societies of Echocardiography and, according to previously published guidelines, including the newly proposed TR grading scale [13, 14]. The procedures are described in detail elsewhere [15, 16].

This study complies with the Declaration of Helsinki. Data collection was approved by the local ethics committee, and written informed consent was obtained from every patient (Local ethic committee number: 2019-537; NCT04559256).

### Cardiopulmonary exercise testing

CPET was performed using a workload controlled upright cycle ergometer. The same equipment was used for baseline and follow-up examinations. CPET was performed pursuant to current German guidelines [17]. A detailed CPET and also CWRET protocol is given in the supplemental section. CPET was continued up to maximum symptom-limited tolerance, or until occurrence of ECG changes suggestive of acute myocardial ischemia, arrhythmias or a sudden drop in blood pressure or oxygen saturation during exercise. Key parameters and threshold values were determined according to current recommendations [17, 18].

### Constant work exercise testing

CWRET (Fig. 1) was performed after an appropriate recovery time from the CPET (at least 4 h). After 3 min of unloaded cycling, the work rate was abruptly increased to 75% of the peak work rate in the initial incremental test. The same termination criteria as for the incremental CPET were applied. The time from the initial increase in work rate up to test termination was recorded. The ECG was monitored continuously, recordings of the ECG and blood pressure were performed at least every 2 min. Ventilation and gas exchange were also continuously measured on a breath-by-breath basis. For the analysis, the gas exchange data were averaged by 10-s time frames.

### Statistical analysis

Statistical analysis was performed using the SPSS-Software suite (Version 22, IBM Corporation, Armonk, NY, USA). Given the small sample size, continuous were presented as median and interquartile range (IQR). Categorical variables are presented as frequencies and percentages. Student’s *t* test was performed for unpaired and paired parametric samples, their analogs for nonparametric samples (Mann–Whitney and Wilcoxon signed rank) or the Chi-square test and McNemar’s were performed for group comparisons, when appropriate. For analysis of collinearity, Pearson’s and Spearman’s correlation coefficients were calculated, as appropriate. *p* < 0.05 was considered to indicate statistical significance.

## Results

### Baseline characteristics

Thirty patients (16 female, 14 male) with complete data acquisition were included into the final analysis. On average, patients presented with a moderate surgical risk (EuroSCORE II 5.6 ± 4.5%). Patients’ median age was 80.5 [74.8; 82.3] years. A high rate of atrial fibrillation (90.0%) was present. On average, mean pulmonary artery pressure was 24.8 ± 6.3 mmHg. Nine patients (26.7%) had an impairment of RV function according to Dietz et al. (tricuspid annular plane systolic excursion [TAPSE] < 17 mm tricuspid valve ring diameter ≥ 40 mm) [19] and ten patients (33.3%) had RV function impairment according to Brener et al. (TAPSE/PAPsys < 0.406) [20]. Detailed baseline characteristics are provided in Table 1.

**Table 1:** Baseline characteristics of the study group

Characteristics (*n* = 30)	
Age	80.5 [74.8; 82.3]
Female	53.3% (16)
Body mass index [kg/m^2^]	24.3 [23.0; 28.8]
EuroScore II (%)	4.2 [2.6; 7.4]
TriScore	4 [4; 4]
Atrial fibrillation	90.0% (27)
Diabetes mellitus	26.7% (8)
Chronic obstructive pulmonary disease	13.3% (4)
Coronary artery disease	43.3% (13)
Stroke	13.3% (4)
History of myocardial infarction	3.4% (1)
History of cardiac surgery	43.3% (13)
Pacemaker lead through tricuspid valve	23.3% (7)
Dialysis	∅
NTpro-BNP [pg/ml]	1830.0 [898.3; 3152.5]

*NTpro-BNP* amino terminal pro-brain natriuretic peptide

### Procedural and clinical outcome

Technical success was achieved in all cases. TR reduction ≥ 2 grades was achieved in 93.3% of the patients (TR grade ≤ moderate at discharge in 83.3% of patients). There were no serious complications (e.g., device detachment, cardiac structural complications (including leaflet damage or perforation), bleeding requiring transfusion or intervention, cerebrovascular events, renal failure or need for pacemaker). Nine patients underwent transcatheter edge-to-edge repair (TEER). In six cases, the PASCAL system was used, while in three cases, TEER was performed using TriClip devices. In 21 patients, direct annuloplasty with the Cardioband system was performed.

Mean duration of hospitalization was 7.1 ± 2.3 days. During the follow-up period, two patients (6.7%) were re-hospitalized due to recurrent cardiac decompensation and required intensified diuretic therapy. Patients described a significant improvement in functional (NYHA) class (from 93.3% NYHA class ≥ III to 73.3% NYHA class ≤ II, *p* < 0.001) as well as in QOL (MLHFQ-Score: from 33.0 [21.0; 48.0] to 22.5 [16.5; 38.5]; *p* = 0.001). In addition, exercise capacity with regard to the 6-min walking test significantly improved (from 290.0 m [215.0; 340.0] to 315.0 m [260.0; 400.0]; *p* = 0.001).

### Echocardiographic examinations revealed right heart remodeling and improved hemodynamics

Time to follow-up examination was 87.5 [56.5; 153] days. Residual TR remained stable during the follow-up period. The post-procedural transtricuspid gradient was acceptable (1.0 mmHg [1.0; 2.0]) and also remained stable during the follow-up period (*p* = 0.67).

**Fig. 2:**
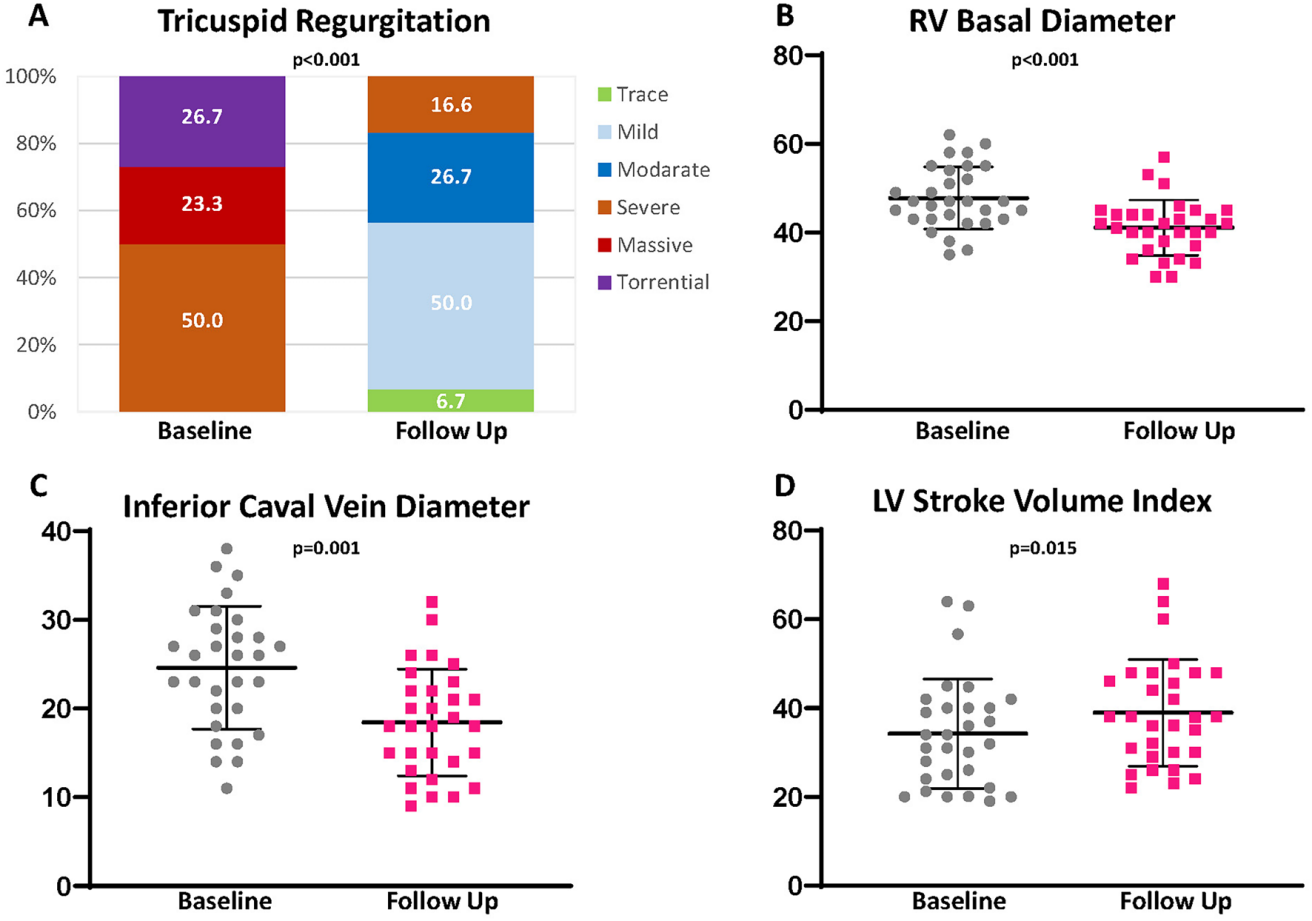
Transcatheter tricuspid valve intervention resulted in stable reduction of tricuspid regurgitation (**A**) with right ventricular remodeling (**B**), decreased right heart congestion (**C**), and more efficient cardiac output (**D**). *LV* left ventricular, *RV* right ventricular

RV basal diameter as well as the diameter of the inferior caval vein decreased significantly. In contrast, left ventricular stroke volume significantly increased (Fig. 2). Regarding functional echocardiographic parameters, RV fractional area change did not show significant differences whereas TAPSE, which reflects the longitudinal shortening of the RV, showed a significant alteration on average. Detailed echocardiographic parameters are provided in Table 2.

**Table 2:** Comparison of baseline and follow-up echocardiographic imaging parameters

Imaging parameters	Baseline	Follow up	*p* value
LV ejection fraction [%]	54.0 [50.0; 62.0]	56.5 [51.0; 61.0]	0.96
LV stroke volume index [ml]	33.0 [23.5; 40.5]	37.9 [29.5; 48.0]	**0.015**
RV basal diameter [mm]	47.0 [43.0; 54.3]	41.5 [36.8; 48.0]	** < 0.001**
RV-FAC [%]	46.0 [39.5; 54.4]	43.3 [33.7; 50.0]	0.18
TAPSE [mm]	19.0 [16.0; 22.0]	16.0 [14.8; 18.0]	** < 0.001**
PAPsys [mmHg]	40.0 [32.0; 48.0]	35.0 [28.5; 45.5]	0.38
TR grade			
Trace	∅	6.7% (2)	** < 0.001**
Mild	∅	50.0% (15)	
Moderate	∅	26.7% (8)	
Severe	50.0% (15)	16.6% (5)	
Massive	23.3% (7)	∅	
Torrential	26.7% (8)	∅	
TR vena contracta [mm]	12.5 [8.0; 15.0]	3.0 [2.0; 5.3]	** < 0.001**
TR-EROA [mm^2^]	50.0 [40.0; 80.0]	10.0 [8.5; 20.0]	** < 0.001**
TR regurgitant volume [ml]	54.0 [39.8; 73.5]	12.5 [6.0; 24.3]	** < 0.001**
TV coaptation gap [mm]	3.0 [2.0;7.0]	1.0 [0.0; 1.0]	**0.013**
TV tethering area [cm^2^]	1.4 [1.0; 2.0]	0.8 [0.5; 1.2]	**0.004**
Vena cava inferior diameter [mm]	26.0 [19.5; 29.3]	18.0 [13.5; 22.5]	** < 0.001**
Appropriate inspiratory vena cava collapse	40.0% (12)	83.3% (25)	**0.001**
Systolic hepatico-venous reflux	83.3% (25)	23.3.0% (7)	** < 0.001**
Transtricuspid gradient [mmHg]	∅	1.0 [1.0; 2.0]	∅

Parameters with significant changes are highlighted in bold

*EROA* effective regurgitation orifice area, *FAC* fractional area change, *LA* left atrial, *LV* left ventricular, *RA* right atrial, *RV* right ventricular, *PAPsys* systolic pulmonary artery pressure, *TAPSE* tricuspid annular plane systolic excursion, *TR* tricuspid regurgitation, *TV* tricuspid valve

### Results of CPET and CWRET

CPET at baseline revealed a severely impaired cardiopulmonary exercise capacity. Patients showed an acceptable motivation and effort with an average peak respiratory coefficient or respiratory exchange ratio (RER) of 1.1 [0.9;1.1].

**Fig. 3:**
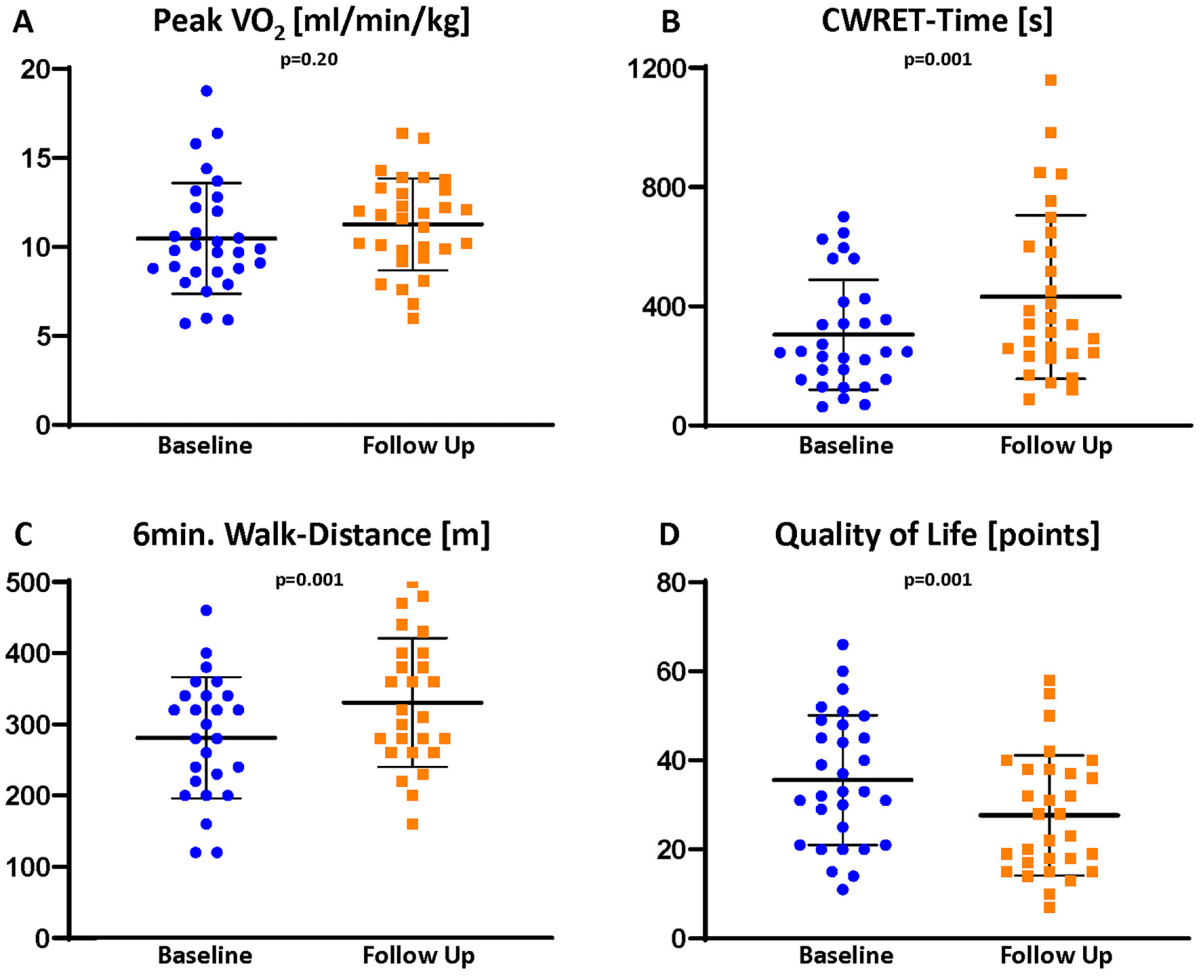
Three months after transcatheter tricuspid valve intervention maximum oxygen consumption (peak *V*O_2_) in cardiopulmonary exercise testing (**A**) does not differ significantly. Rather, the constant work exercise-time (CWRET) testing (**B**), 6-min walking distance (**C**), and quality of life (**D**) significantly increased (assessed by the Minnesota Living with Heart Failure Questionnaire)

**Fig. 4:**
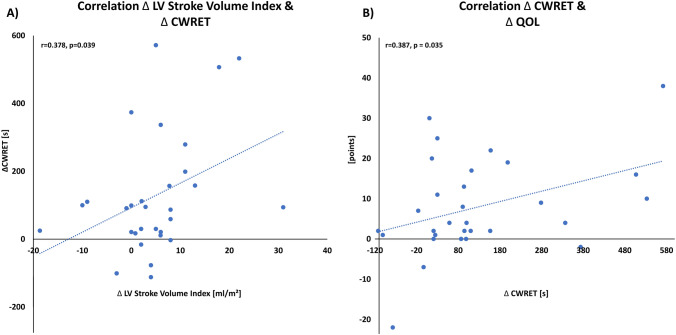
Improvement in constant work-rate exercise-time (CWRET) correlates with increased cardiac output (**A**) and quality of life (QOL) (**B**). *LV* left ventricular

Peak oxygen consumption (peak *V*O_2_) per bodyweight was severely reduced (peak *V*O_2_ 9.9 ml/min/kg [8.6; 12.4]) (Fig. 3). Accordingly, the ventilatory anaerobic threshold (*V*T_1_) was severely impaired (8.5 ml *V*O_2_/min [6.9;10.0]; percent of predicted 50.0% [38.0; 63.5] vs. 50.0% [37.0; 65.0]; *p* = 0.41). Peak oxygen pulse (7.5 ml/beat [6.0; 9.1]) as well as ventilatory equivalents for carbon dioxide (*V*E/*V*CO_2_)-slope (37.0 [32.5; 39.5]) showed major impairments. At follow-up, a trend toward an improved peak *V*O_2_, but no significant improvement of CPET parameters could be observed. Detailed CPET parameters are provided in Table 3. However, CWRET revealed a significant improvement in SEC (exercise duration time from 246.5 s [153.8; 416.8] to 338.5 s [238.8; 611.8]; *p* = 0.001) (Fig. 3). Furthermore, collinearity analysis revealed that, in contrast to the 6-min walking distance (*r* = 0.213; *p* = 0.40 for QOL and *r* = 0.217; *p* = 0.31 for left ventricular stroke volume index), improvement in CWRET time correlated moderately with a reduction in tricuspid effective regurgitant orifice area (EROA; *r* = 0.385; *p* = 0.036), increased left ventricular stroke volume index (*r* = 0.378; *p* = 0.039), increased 6-min walking distance (*r* = 0.502; *p* = 0.009), and improved QOL (*r* = 387; *p* = 0.035) (Fig. 4).

**Table 3:** Comparison of baseline and follow-up results in cardiopulmonary exercise testing (CPET)

Exercise parameters	Baseline	Follow up	*p* value
Maximal work rate [Watt]	57.0 [44.5; 66.5]	58.0 [49.0; 75.5]	0.17
*V*O_2_ at rest [ml/min]	260.0 [200.0; 280.0]	230.0 [200.0; 290.0]	0.65
*V*O_2_ at rest per body weight [ml/min/kg]	3.3 [2.9; 4.1]	3.2 [2.9; 3.9]	0.64
Peak *V*O_2_ [ml/min]	740.0 [570.0; 885.0]	830.0 [640.0; 912.5]	0.15
Peak *V*O_2_ per body weight [ml/min/kg]	9.9 [8.6; 12.4]	11.7 [9.7; 13.3]	0.27
Peak *V*O_2_ (percent of predicted) [%]	59.5 [46.8; 67.8]	62.5 [51.8; 74.0]	0.20
*V*O_2_ at anaerobic threshold (*V*T_1_) [ml/min]	610.0 [510.0; 680.0]	600.0 [560.0; 790.0]	0.42
*V*T_1_ per body weight [ml/min/kg]	8.5 [6.9;10.0]	9.3 [7.9; 11.0]	0.44
*V*T_1_ (percent of predicted) [%]	50.0 [38.0; 63.5]	50.0 [37.0; 65.0]	0.41
Heart rate at rest [min^−1^]	70.0 [62.0; 80.0]	75.0 [60.0; 87.5]	**0.038**
Peak heart rate [min^−1^]	98.0 [86; 114.5]	104.0 [87; 117]	0.53
Blood pressure systolic at rest [mmHg]	110.5 [99.3; 125.8]	113.0 [102.8; 124.0]	0.48/
Blood pressure diastolic at rest [mmHg]	68.0 [60.0; 77.0]	70.0 [56.0; 79.0]	0.97
Peak Blood pressure systolic [mmHg]	133.0 [121.0; 141.0]	135.0 [114.3; 151.5]	**0.013**
Peak Blood pressure diastolic [mmHg]	73.0 [61.0; 77.5]	67.8 [59.0; 78.8]	0.86
Minute ventilation at rest [l/min]	10.5 [9.0; 12.0]	11.0 [8.0; 14.0]	0.43
Peak minute ventilation [l/min]	30.0 [23.5; 35.0]	34.0 [28.5; 40.0]	0.40
*V*E/*V*CO_2_-slope	37.0 [32.5; 39.5]	36.0 [33.0; 39.0]	0.56
*V*E/*V*CO_2_ at *V*T1	38.0 [31.0; 40.0]	36.0 [33.0; 39.0]	0.62
*V*E/*V*CO_2_ nadir	30.0 [28.5; 36.0]	34.0 [30.0; 36.5]	0.27
PETCO_2_ at *V*T1 [mmHg]	31.0 [29.0; 36.0]	32.0 [29.0; 35.0]	0.41
Peak PETCO_2_ [mmHg]	33.0 [31.5; 37.0]	33.0 [31.0; 36.0]	0.41
Oxygen pulse at resting [ml/beat]	3.7 [2.8; 4.0]	2.8 [2.5; 4.1]	0.12
Peak oxygen pulse [ml/beat]	7.5 [6.0; 9.1]	7.6 [6.4; 9.4]	0.31
RER at rest	0.9 [0.8; 0.9]	0.9 [0.8; 1.0]	0.45
Peak RER	1.1 [0.9;1.1]	1.1 [0.9;1.1]	0.39
CWRET [s]	246.5 [153.8; 416.8]	338.5 [238.8; 611.8]	**0.001**
New York Heart Association class			
I	∅	3.3% (1)	** < 0.001**
II	6.7% (2)	70.0% (21)	
III	93.3% (28)	26.7% (8)	
IV	∅	∅	

Parameters with significant changes are highlighted in bold

*CWRET* constant work-rate exercise time; *PETCO2* end-tidal carbon dioxide partial pressure; *RER* respiratory coefficient or respiratory exchange ratio; *VE* pulmonary ventilation; *VE/VCO*_*2*_ ventilatory equivalents for carbon dioxide; *VO*_*2*_ oxygen consumption; *VT*_*1*_ ventilatory anaerobic threshold

Pre-existing impairment of right ventricular function defined by Brener et al. or Dietz et al. did not significantly affect the procedural results or results in exercise tests (Supplemental Table 1). Importantly, there were no significant differences in baseline structural or functional parameters or procedural results between the TEER and annuloplasty groups (Supplemental Tables 2, 3, and 4).

## Discussion

To our knowledge, this is the first prospective evaluation of CPET and CWRET in TR patients undergoing TTVI. The main findings are fourfold. First, CPET revealed a severely impaired cardiopulmonary exercise capacity in our cohort of severe TR patients. Second, TTVI does not result in a statistically significant improvement of peak *V*O_2_ oxygen during exercise. Third, CWRET revealed a significant improvement of SEC regardless of baseline RV function. Finally, this improvement correlates with a reduction of TR parameters, increased left ventricular stroke volume, and QOL.

### Severely reduced peak *V*O_2_ in TR patients undergoing TTVI

Baseline CPET analysis confirmed that the health condition in our cohort of TR patients was severely limited and unsuitable/high risk for surgery. It is known that *V*T_1_ is a valid risk predictor for non-thoracic surgery, with threshold values of lower than 9–12 ml *V*O_2_/kg/min for a worse post-surgical outcome [21, 22]. *V*O_2_ at *V*T_1_ is well below this threshold in our cohort. Interestingly, after TTVI patients did not present with a significant detectable improvement in maximum exercise capacity, despite significant functional improvements such as NYHA class, 6MWD and QOL. The underlying reason may be multifactorial and remains to be elucidated [23]. Pathophysiological considerations on the critical power concept suggest that treatment effects may be first seen in the submaximal endurance part of the power–duration curve (Fig. 1), and less pronounced in maximal exercise capacity [10]. This is in line with mixed results from previous trials in heart failure and pulmonary arterial hypertension that have used peak *V*O_2_ as a measure of treatment effects [11, 24]. In addition, there is published data from a previous retrospective analysis of functional improvement after interventional valve repair showing an improvement in peak *V*O_2_ at 3 months [25]. However, the respective patient cohort was younger and probably more impaired before the procedure, as the average peak *V*O_2_ at baseline was lower than in our patients. Interestingly, the absolute peak *V*O_2_ values at follow-up were comparable to our results. TR patients undergoing TTVI are generally elderly patients with several comorbidities which may also negatively affect exercise capacity. Considering the left ventricle, Benito-Gonzáles et al. reported that transcatheter mitral valve repair in patients with severe mitral regurgitation may result in an improvement in maximum oxygen uptake 6 months post-procedurally (9.8 [9.1; 13.4] ml/kg/min vs. 13.5 [12.1; 16.8] ml/kg/min; *p* = 0.033) [26]. This study comprised a very small cohort consisting of 11 patients while explicitly excluding patients with advanced age and severe comorbidities. This may be an additional explanation for the varying results. Furthermore, besides the hemodynamic dimension, exercise capacity also depends on the muscular and cellular oxidative systems. Patients suffering from the aforementioned comorbidities certainly do not often exercise on maximum levels in their daily lives. This could explain that improvements in peak *V*O_2_ were not significant due to chronic muscular deconditioning. Whether or not a customized exercise regimen could increase peak *V*O_2_ should be part of future prospective trials.

### Increase in constant work-rate exercise test time

On the other hand, endurance time at constant work rate (CWRET) increased significantly after TTVI. As predicted by the critical power concept, this confirms that submaximal endurance capacity increases in this patient cohort before any relevant changes in maximal exercise capacity may be visible [27].

The fractional improvement in SEC, which is more likely to reflect daily life exercise challenges, was much greater and therefore detected more easily than any improvement in peak *V*O_2_. The 6MWD, which also reflects a self-paced maximal sustainable power [27], also shows a significant improvement, as expected. However, in contrast to the 6MWD, improvements in SEC correlate with a reduction in TR (TR-EROA), improved cardiac output, and improved QOL.

Despite the aforementioned advantages of CWRET, this method is more complex, needs more clinical resources, and suffers from limited patient compliance, compared to the 6MWD. Patients have to be motivated to undergo a preliminary incremental ramp CPET to determine an individual constant work rate which may also be time-consuming for patients and clinicians. Nevertheless, little is known about the causes and severity of exercise intolerance in these individuals. Since CWRET provides a more detailed insight in patients’ submaximal exercise capacity, it may represent the most appropriate tool to quantify clinical improvements after TTVI, preserving the acquisition of metabolic exercise data by gas exchange measurements and providing insights into potential mechanisms of exercise limitation. Even though the improvement of CWRET is of interest, the lack of improvement in peak *V*O_2_ is another meaningful finding, supporting the importance of other, non-TR-related mechanisms of exercise limitation in these patients.

### Clinical implications

The TRILUMINATE pivotal trial has shown that TTVI appears to have clinical rather than prognostic benefit in patients with severe TR and improves QOL [28]. Given the high morbidity and advanced age of this patient population, the clinical benefit might be of greater importance to patients, possibly even more than survival benefit.

With that in mind, CWRET might be a sensitive examination to assess functional improvement in patients undergoing TTVI and could help elucidate non-TR-related mechanisms of exercise limitation, including chronic muscular deconditioning. Based on these findings, individual treatment strategies of comorbidities and exercise prescription may be optimized.

Some studies have shown that in advanced heart failure, exercise training does not appear to have a significant effect on exercise capacity in the maximal exercise range [29]. Whether exercise training can achieve improvements in the submaximal range needs to be investigated in future studies.

## Limitations

Several limitations apply to our study. First, our study is clearly limited by its modest sample size. Hence, we cannot rule out insufficient power to detect hitherto hidden associations. Second, due to the sample size, the study is descriptive in nature, hypothesis-generating and not designed to explain the mechanisms behind the observed phenomena. Third, determining the critical power in patients actually requires several constant work-rate tests. Instead, due to feasibility, we used a constant work rate at 75% of maximal exercise capacity, as proposed by Malaguti et al. and Casaburi et al. [27, 30]. In addition, our study is non-randomized and unblinded. Submaximal exercise capacity is an objective, however, a patient-dependent outcome even though patient proved adequate motivation and effort assessed with peak RER. Patient-reported outcomes are generally more susceptible to the placebo effect than observer-reported outcomes. Moreover, detection and performance bias cannot be fully excluded as outcome assessors were also unblinded. Finally, the follow-up period is only limited to 3 months. It is not within the scope of this study to evaluate whether SEC could act as an independent prognostic predictor in patients undergoing TTVI.

## Conclusion

Submaximal exercise endurance capacity is significantly increased in patients with severe TR undergoing TTVI, reflecting an improvement in QOL especially regarding daily life activities. These improvements correlate with improved hemodynamics and an increased cardiac output.

## Supplementary Information

Below is the link to the electronic supplementary material.

Supplementary file1 (DOCX 66 KB)

## Data Availability

The raw data supporting the conclusions of this article will be made available by the authors on request.

## Abbreviations

6MWD: Six-minute walking distance
CPET: Cardiopulmonary exercise test
CWRET: Constant work-rate exercise test
EF: Ejection fraction
FAC: Fractional area change
LV: Left ventricular
MLHQ: Minnesota Living with Heart Failure Questionnaire
NT-proBNP: Amino terminal pro-brain natriuretic peptide
NYHA: New York Heart Association
PAPsys: Systolic pulmonary artery pressure
PETCO_2_: End-tidal carbon dioxide partial pressure
QOL: Quality of life
RER: Respiratory coefficient or respiratory exchange ratio
RV: Right ventricle
RVF: Right ventricular function
TAPSE: Tricuspid annular plane systolic excursion
TEER: Transcatheter edge-to-edge repair
TR: Tricuspid regurgitation
TTE: Transthoracic echocardiography
TTVI: Transcatheter tricuspid valve intervention
*V*E: Minute ventilation
*V*E/*V*CO_2_: Ventilatory equivalents for carbon dioxide
*V*O_2_: Oxygen consumption
*V*T_1_: Ventilatory anaerobic threshold
