# Supplementary material for: Exercise testing in patients with tricuspid regurgitation undergoing transcatheter tricuspid valve intervention
Source: Clin Res Cardiol. 2024 Oct 9;114(2):261–71. doi: 10.1007/s00392-024-02554-8 (PMC11839858; doi:10.1007/s00392-024-02554-8)
Supplement: Supplementary file 1 — Supplementary file1 (DOCX 66 KB) [file 392_2024_2554_MOESM1_ESM.docx]

Supplemental Table 1: Comparison of right ventricular delta strain values in patients with and without signs of pre-interventional right ventricular dysfunction. TTVI resulted in remarkable RV-Remodeling regardless of initial assumed RV dysfunction

|  | According to Dietz et al. (TAPSE<17mm; TV ring≥40mm) (1) | | | According to Brener et al (2) | | |
| --- | --- | --- | --- | --- | --- | --- |
| Imaging Parameters  Δ (Baseline – Follow Up)) | **Preserved RVF (N=21)** | **Impaired RVF (N=9)** | **p-value** | **TAPSE/PASP ratio ≤0.406 (N=10)** | **TAPSE/PASP ratio >0.406 (N=20)** | **p-value** |
| TR reduction | 2.0 [2.0;3.0] | 2.0 [2.0; 3.0] | 0.97 | 2.0 [2.0; 3.0] | 2.0 [2.0; 3.0] | 0.73 |
| TR vena contracta [mm] | 8.0 [5.5;10.0] | 10.0 [5.5; 13.8] | 0.20 | 8.0 [6.4; 10.0] | 10.0 [6.0; 13.0] | 0.10 |
| TR EROA [mm^2^] | 40.0 [36.5; 50.0] | 45.0 [10.0; 67.5] | 0.96 | 45.0 [15.0; 55.0] | 40.0 [36.5; 65.0] | 0.70 |
| RV basal diameter [mm] | 4.0 [1.5; 7.0] | 9.5 [4.3; 150.0] | 0.12 | 4.5 [2.8; 10.8] | 7.0 [2.5; 10.5] | 0.96 |
| RV-FAC [%] | 0.2 [-4.9;16.7] | 7.1 [-5.3; 18.5] | 0.91 | 9.2 [-21.5; 20.9] | 0.2 [-4.6; 12.5] | 0.89 |
| TAPSE [mm] | 3.0 [1.0; 5.0] | 0.0 [-0.8; 1.8] | **0.005** | 1.5 [-0.3; 3.5] | 3.0 [1.0; 5.5] | 0.16 |
| Vena cava inferior diameter [mm] | 6.0 [2.5; 9.8] | 6.5 [1.8; 11.0] | 0.79 | 8.0 [4.0; 16.0] | 5.0 [2.5; 9.5] | 0.14 |
| LV stroke volume index [ml] | 9.5 [3.5; 14.2] | 2.5 [-6.8; 5.8] | 0.06 | 4.0 [-2.3; 10.3] | 8.0 [0.5; 14.5] | 0.28 |
| CWRET [s] | 87.0 [4.0; 178.5] | 97.5 [23.3; 280.3] | 0.55 | 97.0 [-21.5; 168.3] | 87.0 [19.0; 218.0] | 0.75 |
| QOL Score | 8.5 [1.3; 18.3] | 11.0 [4.0; 11.0] | 0.27 | 40.0 [20.0; 80.0] | 40.0 [5.0; 97.5] | 0.77 |
| Six min. walked distance [m] | 40.0 [15.0; 92.5] | 40.0 [30.0; 150.0] | 0.62 | 9.5 [-10.8; 26.0] | 9.0 [4.0; 14.5] | 0.99 |

Term explanations:

Supplemental Table 2: Comparison of echocardiographic and clinical delta values in edge-to-edge and annuloplasty approaches

| Imaging Parameters  Δ (Baseline – Follow Up)) | TEER  (N=9) | Annuloplasty  (N=21) | p-value |
| --- | --- | --- | --- |
| TR reduction | 2.0 [2.0; 3.0] | 2.0 [2.0; 2.0] | 0.07 |
| TR vena contracta [mm] | 6.0 [5.0; 11.5] | 8.0 [6.5; 10.0] | 0.60 |
| TR EROA [mm^2^] | 40.0 [15.0; 60.0] | 50.0 [40.0; 55.0] | 0.22 |
| RV basal diameter [mm] | 7.0 [2.0; 12.5] | 5.0 [3.0; 9.5] | 0.99 |
| RV-FAC [%] | -2.7 [-4.9; 8.6] | 7.0 [-9.1; 21.2] | 0.68 |
| TAPSE [mm] | 3.5 [1.3; 7.0] | 2.0 [0.0; 4.5] | 0.12 |
| Vena cava inferior diameter[mm] | 7.0 [-0.5; 9.5] | 6.5 [4.0; 9.8] | 0.67 |
| LV stroke volume index [ml] | 11.0 [7.4; 22.3] | 5.0 [-3.5; 8.0] | 0.20 |
| CWRET [s] | 112.0 [55.5; 353.0] | 59.0 [9.0; 134.0] | 0.20 |
| QOL Score | 16.5 [10.8; 19.8] | 40.0 [-10.0; 110.0] | 0.11 |
| Six min. walked distance [m] | 50.0 [25.0; 82.5] | 50.0 [25.0; 82.5] | 0.84 |

Term explanations: FAC= fractional area change, RA= right atrial, RV= right ventricular, RVOT= right ventricular outflow tract, TAPSE= tricuspid annular plane systolic excursion

Supplemental Table 3: Comparison of baseline and follow up echocardiographic imaging parameters separately for the two modalities TEER and Cardioband.

|  | Cardioband (N=21) | | | TEER (N=9) | | |
| --- | --- | --- | --- | --- | --- | --- |
| Imaging Parameters | Baseline | Follow Up | p-value | Baseline | Follow Up | p-value |
| LV ejection fraction [%] | 53.0 [50.0; 62.0] | 56.0 [50.5; 60.5] | 0.66 | 57.0 [51.0; 63.5] | 58.0 [53.0; 65.0] | 0.60 |
| LV stroke volume index [ml] | 31.0 [25.0; 36.0] | 36.0 [28.0; 48.0] | **0.023** | 30.1 [20.7; 35.6] | 37.9 [30.0; 47.0] | **0.011** |
| RV basal diameter [mm] | 45.0 [42.5; 53.0] | 40.0 [34.0; 44.5] | **<0.001** | 47.0 [45.5; 56.5] | 42.0 [41.0; 44.5] | **0.009** |
| RV-FAC [%] | 47.0 [39.1; 56.7] | 43.0 [32.8; 50.0] | 0.19 | 42.0 [37.5; 47.6] | 44.3 [33.8; 48.7] | 0.78 |
| TAPSE [mm] | 18.0 [15.0; 21.5] | 16.0 [14.5; 17.0] | **0.002** | 20.0 [18.3; 24.0] | 17.0 [15.0; 19.0] | **0.007** |
| PAPsys [mmHg] | 40.0 [26.5; 48.0] | 34.0 [28.0; 48.5] | 0.62 | 41.0 [34.5; 53.0] | 38.5 [29.5; 43.5] | 0.35 |
| TR grade  Trace  Mild  Moderate  Severe  Massive  Torrential | ∅  ∅  ∅  47.6% (10)  23.8% (5)  28.6% (6) | 4.8% (1)  38.1% (8)  38.1% (8)  19.0% (4)  ∅  ∅ | **<0.001** | ∅  ∅  ∅  55.6% (5)  22.2% (2)  22.2% (2) | 11.1% (1)  77.8% (7)  ∅  11.1% (1)  ∅  ∅ | **<0.001** |
| TR vena contracta [mm] | 13.0 [9.0; 16.0] | 4.0 [2.0; 6.0] | **<0.001** | 12.0 [8.0; 14.5] | 3.0 [2.0; 3.5] | **<0.001** |
| TR EROA [mm²] | 60.0 [45.0; 90.0] | 10.0 [10.0; 25.0] | **<0.001** | 47.0 [35.0; 70.0] | 10.0 [2.0; 11.0] | **<0.001** |
| TR regurgitant volume [ml] | 56.0 [39.5; 76.5] | 12.0 [6.5; 28.0] | **<0.001** | 46.0 [38.2; 65.0] | 13.0 [4.5; 15.0] | **<0.001** |
| TV coaptation gap [mm] | 2.0 [1.0; 9.8] | 0.0 [0.0; 1.0] | **<0.001** | 2.0 [1.0; 3.0] | 0.0 [0.0; 1.0] | **<0.001** |
| TV tethering area [cm2] | 1.4 [0.9; 2.3] | 0.7 [0.5; 1.2] | **0.004** | 1.5 [1.3; 2.0] | 0.9 [0.6; 1.3] | **0.009** |
| Vena cava inferior diameter [mm] | 26.0 [20.0; 31.0] | 19.0 [13.3; 23.8] | **<0.001** | 23.0 [17.5; 27.5] | 18.0 [13.0; 20.5] | **0.026** |
| Appropriate inspiratory vena cava collapse | 28.6% (6) | 81.0% (17) | **<0.001** | 33.3% (3) | 88.9% (8) | **0.010** |
| Systolic hepatico-venous reflux | 85.7% (18) | 28.6% (6) | **<0.001** | 66.7% (6) | 11.1% (1) | **0.003** |
| Transtricuspid gradient [mmHg] | ∅ | 1.0 [1.0; 1.5] | ∅ | ∅ | 1.0 [1.0; 2.0] | ∅ |

EROA=effective regurgitation orifice area, FAC=fractional area change, LA=left atrial, LV=left ventricular, RA=right atrial, RV=right ventricular, PAPsys=systolic pulmonary artery pressure, TAPSE=tricuspid annular plane systolic excursion, TR=tricuspid regurgitation, TV=tricuspid valve

Table 3: Comparison of baseline and follow up results in Cardiopulmonary Exercise testing (CPET) separately for the two modalities TEER and Cardioband.

|  | Cardioband (N=21) | | | TEER (N=9) | | |
| --- | --- | --- | --- | --- | --- | --- |
| Exercise Parameters | Baseline | Follow Up | p-value | Baseline | Follow Up | p-value |
| Maximal Work Rate [Watt] | 55.0 [41.5; 64.0] | 58.0 [49.0; 75.5] | 0.14 | 59.0 [53.8; 72.5] | 57.0 [49.3; 74.0] | 0.85 |
| VO_2_ at rest [ml/min] | 240.0 [200.0; 280.0] | 230.0 [200.0; 290.0] | 0.82 | 280.0 [230.0; 355.0] | 285.0 [232.5; 305.0] | 0.62 |
| VO_2_ at rest per body weight [ml/min/kg] | 3.1 [2.9; 4.1] | 3.2 [2.9; 3.9] | 0.82 | 3.4 [2.9; 4.9] | 3.2 [2.9; 3.9] | 0.60 |
| PeakVO_2_ [ml/min] | 700.0 [565.0; 830.0] | 830.0 [640.0; 912.5] | 0.25 | 810.0 [625.0; 101.0] | 830.0 [640.0; 912.5] | 0.43 |
| PeakVO_2_ per body weight [ml/min/kg] | 9.8 [8.0; 12.4] | 11.7 [9.7; 13.3] | 0.67 | 9.9 [9.4; 13.0] | 12.2 [10.2; 13.6] | 0.43 |
| PeakVO_2_ (percent of predicted) [%] | 59.0 [45.5; 65.0] | 62.5 [51.8; 74.0] | 0.19 | 65.0 [46.5; 80.0] | 70.0 [57.0; 79.5] | 0.33 |
| VO_2_ at anaerobic threshold (VT_1_) [ml/min] | 600.0 [487.5; 637.5] | 600.0 [560.0; 790.0] | 0.43 | 720.0 [590.0; 760.0] | 710.0 [607.5; 857.5] | >0.99 |
| VT_1_ per body weight [ml/min/kg] | 8.1 [6.7; 9.8] | 9.3 [7.9; 11.0] | 0.44 | 9.4 [8.6; 11.6] | 9.6 [8.5; 11.0] | 0.97 |
| VT_1_ (percent of predicted) [%] | 43.5 [36.3; 65.0] | 50.0 [37.0; 65.0] | 0.41 | 64.0 [45.0; 80.0] | 58.5 [44.3; 68.0] | 0.70 |
| Heart rate at rest [min^-1^] | 68.0 [60.1; 81.0] | 75.0 [60.0; 87.5] | 0.053 | 73.5 [69.3; 79.0] | 77.0 [63.0; 92.0] | 0.49 |
| Peak heart rate  [min^-1^] | 98.0 [82.5; 107.0] | 104.0 [87.0; 117.0] | 0.69 | 106.5 [88.8; 134.3] | 124.5 [82.0; 127.5] | 0.16 |
| Blood pressure systolic at rest [mmHg] | 110.5 [96.0; 129.0] | 113.0 [102.8; 124.0] | 0.42 | 110. [102.0; 123.8] | 114.5 [108.3; 127.5] | 0.90 |
| Blood pressure diastolic at rest [mmHg] | 68.0 [60.5; 80.0] | 70.0 [56.0; 79.0] | 0.20 | 67.6 [59.3; 73.0] | 80.0 [70.8; 95.3] | 0**.019** |
| Peak Blood pressure systolic [mmHg] | 133.0 [119.8; 137.05] | 135.0 [114.3; 151.5] | 0.12 | 137.0 [117.0; 143.0] | 158.0 [136.5; 176.5] | 0.053 |
| Peak Blood pressure diastolic [mmHg] | 74.0 [69.8; 77.8] | 67.8 [59.0; 78.8] | 0.50 | 61.0 [59.0; 78.0] | 77.5 [52.5; 92.0] | 0.47 |
| Minute ventilation at rest [l/min] | 10.0 [8.5; 12.0] | 11.0 [8.0; 14.0] | 0.32 | 12.0 [10.0; 15.5] | 13.0 [10.3; 14.8] | 0.87 |
| Peak minute ventilation [l/min] | 29.0 [21.5; 32.0] | 34.0 [28.5; 40.0] | 0.09 | 34.5 [31.8; 35.0] | 39.5 [31.3; 49.5] | 0.22 |
| VE/VCO_2_-Slope | 37.0 [33.0; 40.3] | 36.0 [33.0; 39.0] | 0.29 | 37.5 [30.5; 41.8] | 38.5 [36.5; 42.0] | 0.14 |
| VE/VCO_2_ at VT1 | 37.0 [30.8; 40.3] | 36.0 [33.0; 39.0] | 0.24 | 38.0 [33.0; 40.0] | 38.5 [36.0; 44.0] | 0.37 |
| VE/VCO_2_ nadir | 31.0 [27.0; 35.5] | 34.0 [30.0; 36.5] | 0.66 | 29.5 [29.0; 37.0] | 34.5 [34.0; 40.3] | 0.17 |
| PETCO_2_ at VT1 [mmHg] | 32.0 [28.8; 36.0] | 32.0 [29.0; 35.0] | 0.14 | 31.0 [30.0; 38.0] | 31.5 [28.8; 33.0] | 0.34 |
| Peak PETCO_2_ [mmHg] | 34.0 [31.5; 36.5] | 33.0 [31.0; 36.0] | 0.99 | 33.0 [31.3; 37.8] | 33.0 [30.3; 35.8] | 0.12 |
| Oxygen pulse at resting [ml/beat] | 3.3 [2.8; 4.0] | 2.8 [2.5; 4.1] | 0.12 | 4.2 [2.7; 4.9] | 3.6 [2.7; 4.6] | 0.68 |
| Peak oxygen pulse [ml/beat] | 7.5 [5.8; 8.6] | 7.6 [6.4; 9.4] | 0.32 | 7.8 [6.4; 9.2] | 8.1 [6.2; 10.0] | 0.81 |
| RER at rest | 0.9 [0.8; 1.0] | 0.9 [0.8;1.0] | 0.85 | 0.9 [0.9; 1.0] | 0.9 [0.8; 1.0] | 0.35 |
| Peak RER | 1.1 [0.9; 1.1] | 1.1 [0.9; 1.1] | 0.28 | 1.1 [0.9; 1.1] | 1.1 [1.0; 1.1] | 0.39 |
| CWRET [s] | 231.0 [128.5; 419.5] | 338.5 [238.8; 611.8] | **0.013** | 338.0 [216.5; 469.5] | 385.0 [311.0; 800.5] | **0.019** |
| New York Heart Association class  I  II  III  IV | ∅  4.8% (1)  95.2% (20)  ∅ | ∅  71.4% (15)  28.6% (6)  ∅ | **<0.001** | ∅  11.1% (1)  88.9% (28)  ∅ | 11.1% (1)  66.7% (6)  22.2% (2)  ∅ | **<0.001** |
| Correlation  Δ (Baseline – Follow Up) LV stroke volume index and CWRET | r=0.179 | | 0.44 | r=0.512 | | 0.16 |
| Correlation  Δ quality of life and CWRET | r=0.534 | | **0.013** | r=-0.230 | | 0.55 |
| Correlation  Δ Six-minutes walking distance and CWRET | r=0.631 | | **0.005** | r=0.205 | | 0.63 |

CWR=constant-work rate exercise time; LV=left ventricular; PETCO2=End-tidal carbon dioxide partial pressure; RER=Respiratory coefficient or respiratory exchange ratio; VE=Pulmonary ventilation; VE/VCO2=Ventilatory equivalents for carbon dioxide; VO2=Oxygen consumption; VT1= ventilatory anaerobic threshold;

## Methodology of cardiopulmonary exercise testing

The preferred exercise testing ergometer was a work-load controlled upright cycle ergometer, because work rate and the required O_2_ uptake (VO_2_) can be more accurately controlled than on a treadmill. The same equipment was used for baseline and follow-up measurements. All CPET procedures were performed in accordance with current German guidelines, issued by the German Respiratory Society (“Deutsche Gesellschaft für Pneumologie”, DGP) (3).

### ECG Tracings:

ECG tracings were obtained online during rest, warm-up, incremental exercise, constant-work rate exercise and recovery. At least one printout was obtained during rest, warm-up and recovery. Printouts were obtained every 2 minutes during the incremental exercise phase, and at maximum exercise. For the constant-work rate (CWR) exercise test, printouts were obtained during rest, warm-up, recovery and every 2 minutes during the CWR phase.

### Equipment Preparation:

The equipment was calibrated before each test following the instructions given by the manufacturer of the equipment. The patients’ follow-up tests were scheduled at approximately the same time of day as their initial test. If possible, patients were instructed not to eat a meal two hours prior to testing. However, if the patient were diabetic or hypoglycaemic, a light snack was permissible. Water and medication might be taken as normal.

### Exercise Protocol

Preparation of the patient: The tests were done at least two hours after the last meal and at the same time of the day for earlier studies. Before starting the test, the subject was instructed to sit on the cycle ergometer. The seat height was adjusted so that the subject’s legs were almost maximally extended.

Electrodes for measuring the electrocardiogram and heart rate were placed in positions on the patient, according to on-site practice. Blood pressure was preferably measured with an integrated cuff system. With the subject seated on the cycle ergometer while breathing through a mouthpiece or face mask with a flow or volume transducer and capillary sampling tube to measure O_2_ and CO_2_, as provided by the manufacturer, respiratory gases were measured with a standard metabolic cart equipped with O_2_ and CO_2_ analyzers, and an analyzer that converted flow to volume. Peripheral oxygen saturation was constantly recorded by a pulse oxymeter. The patient’s nose was sealed by a noseclip if a mouthpiece was used. The metabolic cart computer was able to control the work rate of the cycle.

The protocol for the symptom-limited incremental CPET consisted of a 3-minute resting phase, a 3-minute warm-up phase ("O watt") and a constantly increasing workload ramp up to the subjects' maximal tolerance. The work was incremented at the rate of 10, 15 or 20 watts per minute, depending on the subject’s age, gender, size and fitness. The optimal work rate was such that the duration of increasing work rate period was approximately 6 to 12 minutes.

The constant work rate exercise test (CWRET) was always performed after the incremental test, ideally on the following day, but with a sufficient resting time of at least 4 hours after the incremental test. The protocol for the CWRET test consists of a 3-minute resting phase, a 3-minute warm-up phase and a symptom-limited constant workload phase, which was set to a workload of 75% of the peak workload in the initial incremental test. The CWRET phase is followed by a 3-minute recovery phase. The initial testing was performed as follows:

- Resting measurements (incremental test and CWRET): Subject sitting on the cycle ergometer, ventilation and gas exchange were continuously measured. An ECG and blood pressure were recorded during the third minute.
- Unloaded cycling or warm-up (incremental test and CWRET): After the 3-minute resting period, the patient started pedaling with the cycle unloaded (free-wheeling) for three minutes, at about 60 revolutions per minute (rpm) on the unloaded cycle (55-65 rpm were permissive). Maintaining the approximate cycling speed could be assisted by the beat of a metronome or a pedal rate meter. An ECG and blood pressure were recorded during the third minute. Ventilation and gas exchange were continuously measured.
- Increasing work rate exercise (incremental test): After 3 minutes of unloaded cycling, the work rate was constantly increased by 10, 15 or 20 watts/min, depending on the subject's age, sex, size and fitness. The ECG was monitored continuously, recordings of ECG and blood pressure were performed at least every 2 minutes. Ventilation and gas exchange were continuously measured. The patient continued up to maximal tolerance until he/she felt too symptomatic (dyspnea, fatigue or chest pain) to continue, or the patient developed ECG patterns indicative of acute myocardial ischemia, arrhythmia or falling blood pressure despite increasing work rate.
- Constant work rate exercise (CWRET): After 3 minutes of unloaded cycling, the work rate was abruptly increased to 75% of the peak work rate in the initial incremental test. The ECG was monitored continuously, recordings of ECG and blood pressure were performed at least every 2 minutes. Ventilation and gas exchange were continuously measured. The patient continued to maximal tolerance until he/she felt too symptomatic (dyspnea, fatigue or chest pain) to continue, or the patient developed ECG indications suggestive of acute myocardial ischemia, arrhythmia or falling blood pressure.
- Recovery period (incremental test and CWRET): To avoid orthostatic hypotension when stopping exercise, a problem more likely in normal subjects, the subject was encouraged to slowly “rock” the unloaded cycle pedals backward and forward to keep up the venous return during the first 30 seconds of recovery. Blood pressure and ECG were recorded during the second minute of recovery. Ventilation and gas exchange continued to be recorded for three minutes into recovery. The patient was observed for at least 3 minutes or longer as determined by the investigator or examiner, before discharge from the laboratory. The major symptoms experienced by the patient during exercise (for example: dyspnea, fatigue, dizziness, chest pain) were recorded.

### References

1. Dietz MF, Prihadi EA, van der Bijl P et al. Prognostic Implications of Right Ventricular Remodeling and Function in Patients With Significant Secondary Tricuspid Regurgitation. Circulation 2019;140:836-845.

2. Brener MI, Lurz P, Hausleiter J et al. Right Ventricular-Pulmonary Arterial Coupling and Afterload Reserve in Patients Undergoing Transcatheter Tricuspid Valve Repair. Journal of the American College of Cardiology 2022;79:448-461.

3. Meyer FJ, Borst MM, Buschmann HC et al. [Exercise Testing in Respiratory Medicine - DGP Recommendations]. Pneumologie 2018;72:687-731.
